# Relationship between spiritual health, resilience, and happiness among a group of dental students: a cross-sectional study with structural equation modeling method

**DOI:** 10.1186/s12909-022-03243-8

**Published:** 2022-03-16

**Authors:** Saeed Hatami, Hajar Shekarchizadeh

**Affiliations:** 1 School of Dentistry, Isfahan (Khorasgan) Branch, Islamic Azad University, Isfahan, Iran; 2 Department of Community Oral Health, School of Dentistry, Community Health Research Center, Isfahan (Khorasgan) Branch, Islamic Azad University, Isfahan, Iran

**Keywords:** Dental students, Happiness, Health, Resilience, Spirituality

## Abstract

**Background:**

Given the importance of spiritual health, resilience and happiness to encounter challenges facing dental students, we aimed to examine these variables, their relationship and the mediating role of resilience between spiritual health and happiness among a group of Iranian dental students in Islamic Azad University of Isfahan.

**Methods:**

In this cross-sectional study, utilizing a stratified sampling method, 150 volunteer dental students in different academic years filled in the Persian version of Spiritual Well-Being scale, the Persian version of Connor-Davidson Resilience Scale, and Isfahan-Fordyce Happiness Inventory. Independent t-test, Kruskal–Wallis test, and Pearson correlation coefficient served for statistical analysis. In addition, we conducted a Structural Equation Modeling analysis.

**Results:**

The mean age of the students was 23.43 ± 3.11, and 56% were women. The mean score of spiritual health, resilience, and happiness was 89.27 ± 16.69, 90.19 ± 15.03, and 295.17 ± 65.82, respectively. Spiritual health of a great majority of dental students was upper medium or high (87.3%), most of them had high level of resilience (69.3%), and 55.3% reported medium level of happiness. Spiritual health was directly associated with both happiness and resilience (*p* <  0.001). Resilience acted as an intermediary variable between spiritual health and happiness (p <  0.001).

**Conclusions:**

Spiritual health was associated with increased happiness, and this relationship was mediated by resilience. Thus, it is recommended to address the issue of spiritual health to those students with lower scores in order to increase their resilience and their level of happiness.

## Background

Spirituality is a sense of connection to something superior, and it encompasses the search for meaning in life [1]. Spiritual health is a state that enables individuals to spend their daily life in a way that leads to understanding their full potential and the meaning and purpose of life, and also leads to their inner happiness [2]. Spiritual health is a dynamic, expandable, conscious, multidimensional, and universal process [3] which affects physical, mental and social health, and its effects are reflected in individual’s behavior [4]. In order to link the two concepts of health and spirituality within the idea of spiritual wellbeing, it has been suggested by Ellison that spiritual wellbeing is an expression of spiritual health [5, 6]. Fehring et al. also explained that spiritual wellbeing is an indication of spiritual health [5, 7].

Resilience is defined as the process of adapting well in face of adversity, trauma, tragedy, threats or significant sources of stress [8]. Individuals’ ability to return to their original state or to successfully endure adverse conditions is called resilience [9].

Happiness as a mental or emotional state is described by positive emotions ranging from satisfaction to intense pleasure. It refers to the fact that how people experience their quality of life [10]. People consider the meaning of happiness as “being in a state of happiness and joy, or other positive emotions” or “being satisfied with their own life”. In many cases a third component also exist: “the absence of depression, anxiety and other negative emotions” [11].

Spiritual health plays an important role in human health, affects coping strategies and can compensate other aspects of health including mental, physical, and social health [12]. Spiritual persons feel more connected to life, and are more satisfied. They find peace and harmony in everything they do, and believe that God or a superior power is ever-present in their every action. Hence, they are happier [1]. Positive relationship between spiritual health and coping abilities [12], between spiritual health and happiness [13], and between resilience and happiness [14] have been described in previous studies.

Dental students usually face numerous stresses including admission to dental school, competitive training to develop clinical skills, dealing with innate feelings related to doctor-patient relationship, high work pressure, exam stress, limited free time, and stress resulting from complying with clinical requirements. These sources of stress might lead to their health problems, burnout, and development of undesirable habits. Thus, they need to be able to cope with their stress [12, 15].

Resilience, as a process of adaptation to life changes, is a protective factor against psychological damage and mental disorders [15]. Spirituality acts as an important source of adaptation, and spiritual health improves individuals’ physical and psychological health, and their quality of life [16]. Spiritual health increases individual’s coping skills, thus it can help dental students, as future health care providers, to overcome critical situations [12]. According to Kamthan et al. [17], believing in a superior power is helpful to achieve inner happiness. Moreover, according to Aboalshamat et al. [14], students with high resilience are happier and more satisfied with their life.

Given the importance of spiritual health, resilience and happiness to encounter challenges facing dental students, we investigated the level of spiritual health, resilience, and happiness among a group of dental students. In addition, the aim of our study was to evaluate the relationship between these three variables, and to examine the effect of spiritual health on students’ resilience and happiness, as well as the mediating role of resilience between the two other variables.

## Methods

We conducted a cross-sectional study on 150 Iranian dental students from Islamic Azad University of Isfahan in 2019. The sample size of 150 was estimated to calculate the simple correlation coefficient between main variables of the study considering the precision of 0.05, power of 80% (β = 0.2), a minimum correlation coefficient of 0.25 (medium effect size) for the significance association in a hypothesis test of *ρ = 0* compared to *ρ* ≠ 0, and considering 20% additional cases due to the possibility of the distortion of questionnaires. This sample is enough for the structural equation modeling method used in the present study as explained by Keline [18].

Applying a stratified sampling method, students from different academic years entered the study. In addition to questions regarding their demographic characteristics (gender, age and academic year), volunteer students were asked to fill in three self-administered questionnaires in order to measure their spiritual health, resilience, and happiness. We used the Persian version [19] of Spiritual Well-Being Scale [20] which has two subscales: Existential Well-Being and Religious Well-Being. Each subscale comprises 10 items on a six-point Likert scale rating from 1 (strongly disagree) to 6 (strongly agree). Reverse scores were considered for questions number 3, 4, 7, 8, 10, 11, 14, 15, 17, 19 and 20. Spiritual Well-Being Scale for each participant ranges from 20 to 120. Scores were then categorized as: low spiritual health (20–40), lower medium spiritual health (41–70), upper medium spiritual health (71–99), and high spiritual health (100–120) [19]. Among our study participants, the Cronbach’s alpha for this scale was calculated as 0.929.

We used the Persian version [21] of the Connor-Davidson Resilience Scale [22], which comprises 25 items on a five-point Likert scale rating from 1 (not true at all) to 5 (true nearly all the time). This scale measures five factors including: 1. notion of personal competence, 2. trust in one’s instincts (tolerance of negative affect), 3. positive acceptance of changes and secure relationships, 4. control, and 5. spiritual influences. Connor-Davidson Resilience Scale for each participant ranges between 25 and 125. Scores were then categorized as: low resilience (< 41), moderate resilience (41–83), high resilience (83<). Among our study participants, the Cronbach’s alpha for this scale was calculated as 0.919.

We used the Persian version of Fordyce Happiness Inventory (Isfahan-Fordyce Happiness Inventory) [23], which includes 48 multi-choice items divided into 16 sections, each section with three items. Each item ranges between 0 and 10. The inventory variables include: be more active and keep busy, spend more time socializing, eliminate negative feelings and problems, develop positive optimistic thinking, expressing feeling, lower expectations and aspirations, be yourself, close relationships, get better organized, be present-oriented, value happiness, be productive at meaningful work, stop worrying, work on a healthy personality, develop an outgoing social personality, and overall happiness. Isfahan-Fordyce Happiness Inventory for each participant ranges from 0 to 480. Scores were then categorized as: low happiness (0–170), medium happiness (170–330), and high happiness (330–480). Among our study participants, the Cronbach’s alpha for this scale was calculated as 0.950.

The Islamic Azad University of Isfahan Ethics Committee approved the study (ID: IR.IAU.KHUISF.REC.1398.183). Participation in this study was voluntary. All students provided their informed consent and filled in anonymous questionnaires.

### Statistical analyses

The data were analyzed with the Statistical Package for the Social Science (SPSS for Windows, version 20.0/PC; SPSS, Chicago, IL, USA). The scatter plots verified the linear relationships between the study variables, and the normal distribution of the data was confirmed using Kolmogorov–Smirnov test. Student’s t-test, Kruskal–Wallis test, and Pearson correlation coefficient served for statistical analysis (level of significance < 0.05). Missing data were present in less than 5% of the questionnaires. The mean substitution method was used to deal with these data.

The conceptual model of the study (the relationship between spiritual health and happiness with the mediating role of resilience) was tested by AMOS software using a Structural Equation Modeling method. We analyzed the goodness of fit of the model using the Comparative Fit Index (CFI), Goodness of Fit Index (GFI), Normed Fit Index (NFI), Root Mean Square Error of Approximation (RMSEA), and Relative Chi-Square (χ2/df).

## Results

Among 150 dental students (Response Rate = 94%), 56% were females. The mean age of the students was 23.43 ± 3.11; a majority of whom were between 21 and 25 years of age. The students’ mean score of spiritual health was 89.27 (SD 16.69; range 41–118). The mean score of religious subscale was 44.99 (SD 8.20; range 20–59). The mean score of existential subscale was 44.28 (SD 9.06; range 17–59). Spiritual health among 30.0% of the student was high, among 57.3% was upper medium, and among 12.7% was lower medium. Spiritual health was not significantly associated with students’ gender (*P* = 0.18), age (*P* = 0.95) and their academic year (*P* = 0.39).

The students’ mean score of resilience was 90.19 (SD 15.03; range 34–120). The highest score belonged to the dimension of “notion of personal competence” (29.73 ± 5.68), and the lowest score belonged to the dimension of “spiritual influences” (7.30 ± 2.02) (Table 1). Resilience among 69.3% of the students was high, among 30.0% was medium, and among 0.7% was low. Resilience was not significantly associated with students’ gender (*P* = 0.40), age (*P* = 0.37) and their academic year (*P* = 0.13).

**Table 1:** Resilience and its dimensions among dental students of Islamic Azad University of Isfahan (*n* = 150)

Dimensions	Mean	SD	Minimum	Maximum
Notion of personal competence	29.73	5.68	11	40
Trust in one’s instincts	23.53	4.78	10	35
Positive acceptance of changes and secure relationships	18.59	3.30	5	25
Control	11.04	2.45	3	15
Spiritual influences	7.30	2.02	2	10
Total score of resilience	90.19	15.03	34	120

The students’ mean score of happiness was 295.17 (SD 65.82; range 116–448). The highest score belonged to the dimension of “value happiness” (23.01 ± 5.22), and the lowest score belonged to the dimension of “lower expectations and aspirations” (12.70 ± 6.50) (Table 2). Happiness among 39.3% of the students was high, among 55.3% was medium, and among 5.3% was low. Happiness was not significantly associated with students’ gender (*P* = 0.42) and age (*P* = 0.39). A significant association revealed between the students’ academic year and their happiness scores (*P* = 0.02). Based on the post hoc test, only happiness score among fourth-year students was significantly higher than that of the fifth-year students.

**Table 2:** Happiness and its dimensions among dental students of Islamic Azad University of Isfahan (*n* = 150)

Dimensions	Mean	SD	Minimum	Maximum
Value happiness	23.01	5.22	7	30
Be yourself	20.23	6.39	0	30
Close relationships	20.21	5.70	3	30
Overall happiness	19.84	6.51	0	30
Develop positive optimistic thinking	19.69	5.97	0	30
Work on a healthy personality	19.49	6.73	1	30
Get better organized	19.01	5.87	2	30
Stop worrying	18.65	5.66	3	30
Spend more time socializing	18.59	6.11	0	30
Eliminate negative feelings and problems	18.23	6.34	0	30
Develop an outgoing social personality	18.02	4.22	0	27
Be more active and keep busy	17.48	6.33	0	30
Be present-oriented	16.91	6.64	0	30
Expressing feeling	16.68	6.18	0	30
Be productive at meaningful work	16.43	7.14	0	30
Lower expectations and aspirations	12.70	6.50	0	28
Total score of happiness	295.17	65.82	116	448

A significant correlation existed between the students’ spiritual health and their resilience score (P <  0.001, *r* = 0.50). Moreover, all dimensions of resilience were significantly correlated to both subscales of spiritual health (*P* <  0.05) (Table 3).

**Table 3:** Correlation between dimensions of resilience and spiritual health among dental students of Islamic Azad University of Isfahan (*n* = 150)

Dimensions	Religious health		Existential health		Spiritual health	
	*r*	*P*-value	*r*	*P*-value	*r*	*P*-value
Notion of personal competence	0.382	< 0.001	0.465	< 0.001	0.440	< 0.001
Trust in one’s instincts	0.187	0.022	0.334	< 0.001	0.273	0.001
Positive acceptance of changes and secure relationships	0.387	< 0.001	0.427	< 0.001	0.422	< 0.001
Control	0.403	< 0.001	0.404	< 0.001	0.418	< 0.001
Spiritual influences	0.590	< 0.001	0.628	< 0.001	0.631	< 0.001
Total score of resilience	0.434	< 0.001	0.526	< 0.001	0.499	< 0.001

A significant correlation existed between the students’ spiritual health and their happiness score (*P* <  0.001, *r* = 0.57). Moreover, all dimensions of happiness were significantly correlated to both subscales of spiritual health (*P* <  0.05). Among various dimensions of happiness, “develop positive optimistic thinking” showed the strongest association with spiritual health (*r* = 0.561) and its subscales: religious health (*r* = 0.520) and existential health (*r* = 0.563) (Table 4).

**Table 4:** Correlation between dimensions of happiness and spiritual health among dental students of Islamic Azad University of Isfahan (*n* = 150)

Dimensions	Religious well-being		Existential well-being		Spiritual well-being	
	*r*	*P*-value	*r*	*P*-value	*r*	*P*-value
Be more active and keep busy	0.327	< 0.001	0.441	< 0.001	0.400	< 0.001
Spend more time socializing	0.257	0.001	0.306	< 0.001	0.293	< 0.001
Eliminate negative feelings	0.461	< 0.001	0.452	< 0.001	0.472	< 0.001
Develop positive optimistic thinking	0.520	< 0.001	0.563	< 0.001	0.561	< 0.001
Expressing feeling	0.292	< 0.001	0.417	< 0.001	0.370	< 0.001
Lower expectations	0.231	0.004	0.319	< 0.001	0.287	< 0.001
Be yourself	0.360	< 0.001	0.316	< 0.001	0.348	< 0.001
Close relationships	0.362	< 0.001	0.419	< 0.001	0.405	< 0.001
Get better organized	0.313	< 0.001	0.331	< 0.001	0.333	< 0.001
Be present-oriented	0.436	< 0.001	0.454	< 0.001	0.461	< 0.001
Value happiness	0.273	0.001	0.288	< 0.001	0.290	< 0.001
Be productive at meaningful work	0.454	< 0.001	0.457	< 0.001	0.471	< 0.001
Stop worrying	0.266	0.001	0.385	< 0.001	0.340	< 0.001
Work on a healthy personality	0.187	0.022	0.246	0.002	0.225	0.006
Develop an outgoing social personality	0.340	< 0.001	0.403	< 0.001	0.386	< 0.001
Overall happiness	0.396	< 0.001	0.510	< 0.001	0.472	< 0.001
Total score of happiness	0.509	< 0.001	0.585	< 0.001	0.568	< 0.001

A significant correlation revealed between the students’ resilience score and their happiness score (*P* < 0.001, *r* = 0.71). In addition, most dimensions of happiness showed significant correlation with the dimensions of resilience (*P* < 0.05) except for “value happiness” and “spiritual influences” (*P* = 0.236); and “get better organized” and “spiritual influences” (*P* = 0.093).

**Fig. 1:**
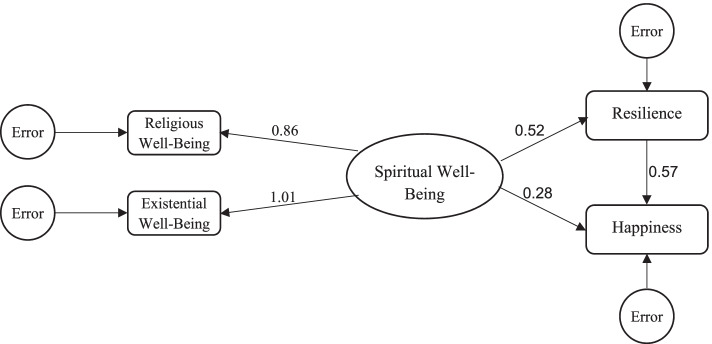
Structural Equation Modeling analysis with the path coefficients of spiritual health, and the mediating role of resilience on happiness

In order to test the relationship between spiritual health and happiness with the mediating role of resilience, we used the Structural Equation Modeling method. The analysis of this model revealed good indices of fitness (RMSEA = 0.065, GFI = 0.990, CFI = 0.91, NFI = 0.998 and χ2/df = 0.7). The direct effect of spiritual health on happiness (standardized coefficient = 0.28, *p* < 0.001), the direct effect of spiritual health on resilience (standardized coefficient = 0.52, *p* < 0.001), and the direct effect of resilience on happiness (standardized coefficient = 0.57, *p* < 0.001) were significant. In addition, the indirect pathway of spiritual health on happiness with the mediating role of resilience was significant (standardized coefficient = 0.296, *p* < 0.001) (Fig. 1).

## Discussion

Spiritual health of a great majority of dental students was upper medium or high (87.3%). This is in line the results of the study by Sen et al. [16] which most Indian medical students (75.7%) reported refined spiritual health. However, in other similar studies on medical, dental or paramedical students, spiritual health of most students was at medium level [12, 13, 24]. The present study was conducted in a dental school in Islamic Republic of Iran which more than 90% of the citizens [25], and a great majority of university attendees are Muslims. As pointed out by Jaberi et al. [25], religious beliefs in Iran as a religious society are closely associated with spiritual health. This might justify the rather high level of spiritual health among our participants.

In our study, spiritual health was not significantly associated with students’ gender, age and their academic year. Results are diverse among Indian students: while Dhama et al. [12] reported no gender difference in spiritual health of dental students, Sen et al. [16] showed higher level of spiritual health among female medical students.

Resilience score of most dental students in our study (69.3%) was high. In contrast, the resilience level of only 8% of students in a dental school in Thailand (72.6%) was higher than normal [26]. Similarly, the resilience of a group of Turkish freshmen health sciences students [27], and a group of medical and dental students in Saudi private colleges was not high [14]. Resilience was not significantly associated with our students’ age, gender, and their academic year. In contrast, Bahadir-Yilmaz et al. [27] reported higher resilience in men than in women, and Aboalshamat et al. [14] reported higher resilience in women than in men. Such difference in findings of the studies seems to be related to the cultural dimension of the concept of resilience. As indicated by Ungar [28], people’s ideologies and beliefs such as gender roles are affected by their culture. Thus, gender differences in resilience should be investigated in different cultures [27].

Happiness score of 55.3% of dental students in our study was medium. The highest score of happiness belonged to the dimension of “value happiness” and the lowest score belonged to the dimension of “lower expectations and aspirations”. Similar to our finding, happiness among a group of Iranian medical sciences students was average [13]. However, happiness level of a group of medical or dental students in Saudi Arabia [14], and a group of Indian medical students [17] was above average. This diversity in the level of happiness between studies might be related to different study populations. Based on a report by Kaipa et al. [10] among Indian dentists, although dentistry is a stressful profession, happiness level of most dentists was higher than average. It seems that factors other than stressful working conditions, might have greater influence on dentists’ level of happiness. For example, factors including demographic characteristics, type of professional attachment, duration and location of the practice, as well as the dentists’ qualification were associated with higher score of happiness among Indian dentists [10].

Similar to a group of Saudi students [14], no significant relationship revealed between dental students’ happiness and their gender. In most previous studies however, happiness was reported to be higher among men than among women [10, 17, 29, 30]. The relationship between happiness and age is also controversial. Similar to the report of Moghadam et al. [29], in our study no significant association existed between dental students’ happiness and their age. In contrast, Kaipa et al. [10] reported a positive correlation between happiness and age among dentists.

In terms of their academic year, happiness score of fourth-year dental students was significantly higher than that of fifth-year students. This might be because fifth year dental students in Iran usually have more clinical requirements, and are more in contact with patients. This situation results to increased stressful conditions which might reflect to their level of happiness. On the contrary, while no significant relationship revealed between medical students’ happiness and their educational level in the study by Moghadam et al. [29], Aboalshamat et al. [14] reported higher happiness among students with higher educational level (internship).

In the present study, we found the direct effect of spiritual health on resilience and happiness, the direct effect of resilience on happiness, and the indirect pathway of spiritual health on happiness with the mediating role of resilience. The significant association between students’ spiritual health and their resilience in our study is in accordance with the results of the study of Indian medical students admitting a significant relationship between spiritual health and adaptive coping skills [16]. According to Dhama et al. [12], spiritual health plays an important role, can compensate for a variety of health conditions such as mental, physical, and social health, and also affects coping strategies in human health.

In line with the study of a group of Iranian medical sciences students [13], a significant association revealed between students’ spiritual health and their happiness. The relationship between happiness and belief in a superior power or having religious beliefs has also been approved in other studies on medical and other health science students: individuals with more religious attitudes reported higher level of happiness [17, 31]. On the contrary, Francis et al. report no relationships between religion and happiness among German students [32]. The significant association between students’ resilience and happiness in our study, is in line with the study of Saudi medical and dental students [14].

The present study investigated the relationship between spiritual health, resilience, and happiness among dental students. To the best of our knowledge, this is the first study examined these three variables simultaneously among dental students. For data collection, we used standard questionnaires: the Persian version of Spiritual Well-Being Scale, the Persian version of Connor-Davidson Resilience Scale, and the Isfahan-Fordyce Happiness Inventory. Validity and reliability of the Persian versions of these questionnaires had been approved in previous studies. High response rate is another strength of the present study. However, questionnaire nature of the study might have resulted to socially acceptable answers. In order to overcome such limitation, we used anonymous self-administered questionnaires. In addition, data collection by three questionnaires might have negatively affected the students’ willingness to participate. We tried to overcome this limitation by explaining the importance of the study for students. Finally, we conducted the study in a single dental school, thus we cannot generalize the findings to all dental students.

## Conclusion

Spiritual health of a great majority of Iranian dental students was upper medium or high. Most students revealed high level of resilience. However, happiness score among half of the students was medium. A significant association revealed between dental students’ spiritual health, resilience, and happiness. Spiritual health was significantly associated with both resilience and happiness. Resilience acted as an intermediary between spiritual health and happiness. Thus, it is recommended to address the issue of spiritual health to those students with lower scores in order to increase their resilience and their ability to cope with the difficulties of their studies resulting to a higher level of happiness.

## Data Availability

The datasets generated during and analyzed during the current study are not publicly available due to our commitment to Islamic Azad University to disseminate the data just in specific cases. Data set is available from the corresponding author on reasonable request.

## Abbreviations

CFI: Comparative Fit Index
GFI: Goodness of Fit Index
NFI: Normed Fit Index
RMSEA: Root Mean Square Error of Approximation
